# Chemical Composition and Biological Activities of the Essential Oils of *Chrysophyllum albidum* G. Don (African Star Apple)

**DOI:** 10.1155/2021/9911713

**Published:** 2021-06-11

**Authors:** Daniel Nartey, Joseph Nana Gyesi, Lawrence Sheringham Borquaye

**Affiliations:** ^1^Department of Chemistry, Kwame Nkrumah University of Science and Technology, Kumasi, Ghana; ^2^Central Laboratory, Kwame Nkrumah University of Science and Technology, Kumasi, Ghana

## Abstract

The volatile compounds of the fruit and leaf essential oils of the African star fruit, *Chrysophyllum albidum* G. Don, were characterized by gas chromatography-mass spectrometry in this study. The antimicrobial, antibiofilm, and antioxidant activities of the essential oils were also investigated. Thirty-five and thirty-four compounds, representing 97.84% and 97.87%, were identified in the leaf and fruit essential oils, respectively. The antimicrobial activity of the oils was evaluated *in vitro* against eight pathogens using the broth microdilution method. The fruit essential oil exhibited broad-spectrum antimicrobial activity in the antimicrobial susceptibility test, with minimum inhibitory concentrations (MICs) ranging from 0.195 to 6.250 mg/mL, while the leaf essential oils showed antimicrobial activity with MICs in the range of 6.875–13.750 mg/mL. The antibiofilm activity was assessed via the crystal violet staining assay, with *Pseudomonas aeruginosa* as the model organism. The concentrations of the leaf and fruit essential oil required for half-maximal inhibition of biofilm formation (BIC_50_) were 6.97 ± 0.56 and 4.78 ± 0.21 mg/mL, respectively. In evaluating antioxidant activity, the total antioxidant capacity obtained from the phosphomolybdenum assay was 104.8 ± 2.4 and 101.6 ± 0.8 *μ*g/g AAE for leaf and fruit essential oils, respectively. The IC_50_ values obtained from the hydrogen peroxide scavenging, 1,1-diphenyl-2-picrylhydrazyl (DPPH) free radical scavenging, and inhibition of lipid peroxidation assays were 301.8 ± 0.7 and 669.2 ± 2.1 *μ*g/mL, 1048.0 ± 0.3 and 1454.0 ± 0.3 *μ*g/mL, and 460.1 ± 2.7 and 457.4 ± 0.3 *μ*g/mL for both leaf and fruit essential oils, respectively. The results obtained in this study suggest that the leaf and fruit essential oil of *Chrysophyllum albidum* G. Don could find potential use in the food, cosmetic, and pharmaceutical industries as preservative and pharmaceutical agents.

## 1. Introduction

Essential oils are common in nature and mostly represent the distinctive flavors and aromas of many plants [1]. Many herbs and spices such as garlic, black cumin, cloves, cinnamon, thyme, bay leaves, mustard, and rosemary have been reported to contain important essential oils [2]. Essential oils have been obtained from various parts of plants–fruits, leaves, roots, stem bark, seeds, etc. Plants utilize essential oils for various purposes. Some essential oils function as integral components of plant defense strategy by acting as insecticidal and antimicrobial agents. In some plants, essential oils play key roles in attracting insects for seed dispersal and flower pollination [3]. Several researches have reported essential oils as possessing a wide range of bioactivities and these activities have been attributed to the constituents of these oils. Essential oil constituents such as limonene, eugenol, pinene, carvone, and linalool have been suggested as agents responsible for the antimicrobial potency of some essential oils. Other essential components such as thymol and menthol elicit antioxidant tendencies whereas aromadendrene and T-cadinol play a role in anti-inflammatory actions [4, 5].

Even though several reports of the antimicrobial activity of essential oils exist in the literature, very few studies center on the route by which these oils exert their antimicrobial action. The ability of the tea tree oil to disrupt the bacterial cytoplasmic membrane and the accompanying loss of chemiosmotic control has been suggested as the most likely source of its lethal action against *Escherichia coli*, *Staphylococcus aureus*, and *Candida albicans* [5, 6]. Other essential oil constituents, such as carvacrol and thyme, have been shown to effect their antimicrobial action by interfering with biofilm formation [7]. Biofilm formation by bacteria affords pathogenic cells the ability to escape the lethal effects of host antimicrobial molecules and systemic antibiotics. Oftentimes, this mechanism developed by pathogens results in antibiotic resistance. Biofilm inhibition or removal is therefore a potentially attractive route for eliminating bacteria [8].

Essential oils have also been explored for their antioxidant properties. Natural antioxidants are in demand due to their perceived safety profiles. Additionally, the use of synthetic antioxidants such as butylated hydroxyanisole (BHA) and butylhydroxytoluene (BHT) have been discouraged due to health, safety, and environmental concerns. As such, there is a need to find alternative antioxidants that do not carry these negative tags [9]. Lipid peroxidation, a direct consequence of the action of free radicals on fats and oils, can easily cause a deterioration in the quality of food and also decrease the nutritional value of food. Lipid peroxidation usually results in offensive flavors and odors of foods. Antioxidants could potentially contribute to the prevention and treatment of diseases caused by radicals and reactive oxygen and nitrogen species. Antioxidants could also be used in food preservation to mop up the radicals that cause lipid peroxidation [10]. Several essential oils have been shown to possess antioxidant activities. Essential oils from the leaf and fruit of *Annona muricata* showed interesting antioxidant capabilities [11].

*Chrysophyllum albidum* (Linn), commonly known as the African star apple, belongs to the family Sapotaceae which has about 800 species. It is primarily a forest tree species, and its natural occurrence has been reported in diverse ecological zones in Nigeria, Uganda, Niger Republic, Cameroon, and Cote d'Ivoire [12]. In Ghana, the tree is usually domesticated while some are found in the forest. The fruit is known as a natural source of antioxidants that can reduce free radical-mediated diseases such as diabetes, cancer, and coronary heart disease [13]. The fruits also contain 90% anacardic acid, which is used industrially in protecting wood and as a source of resin, while several other components of the tree including the roots and leaves are used as a remedy for yellow fever and malaria. The leaves are used as emollients and for the treatment of skin eruptions, diarrhea, and stomachache [14]. Several studies on *Chrysophyllum albidum* have focused on the nutritional content and the biological activities of the crude plant extracts [15, 16]. Essential oils from the African star apple have only been explored to a limited extent. In 2013, Lasekan and coworkers examined the volatile constituents of the fruit pulp of the African star apple that contributed to the unique aroma and flavor of the fruit juice. Four different varieties of the fruits were used [17]. Recently, Ishola utilized hydrodistillation to extract essential oils from various parts of the plant–fruit bark, root bark, stem bark, seed bark, leaf and seed–and evaluated the antioxidant and antimicrobial capabilities of the oils [18]. As expected, different plant varieties yielded essential oils with different compositions. Since the biological activities are based on the identity and amounts of the various chemical constituents in these essential oils, the chemical composition and biological activities of the fruit pulp and leaf essential oils of Ghanaian cultivars of *Chrysophyllum albidum* were studied. Gas chromatography-mass spectrometry was used in identifying the chemical constituents of the essential oils. Biological activities investigated included antioxidant, antimicrobial, and antibiofilm activities.

## 2. Methods

### 2.1. Plant Material

Fresh fruits and leaves were sampled from Kumasi in the Ashanti region, Ghana, in February 2019. Samples were transported to the laboratory at the Department of Chemistry, Kwame Nkrumah University of Science and Technology (KNUST), Kumasi, and stored in a refrigerator at 4°C. Plant identification and authentication were carried out at the Department of Herbal Medicine, KNUST.

### 2.2. Essential Oil Extraction

Fresh fruits were washed with distilled water. Afterwards, the fruit pulp was separated from the fruit bark and seeds, and then subjected to steam distillation for 4 hours in a modified Clevenger-type setup. Fresh leaves were also washed and extracted similarly. The essential oils were recovered and treated with anhydrous sodium sulfate to remove traces of water. Essential oils were stored at 4°C until used in further analysis. The essential oil yields were calculated with respect to the fresh weight of the plant material before distillation (expressed as percentage w/w of the fresh material).

### 2.3. Chemical Composition Analysis Using GC-MS

Chemical composition of the essential oils was assayed on a Perkin Elmer GC Clarus 580 Gas chromatograph interfaced with a Perkin Elmer (Clarus SQ 8 S) mass spectrometer. A DB-5 (ZB-5HTMS; 5% diphenyl/95% dimethylpolysiloxane) fused capillary column with dimensions 30 × 0.25 mm ID × 0.25 *μ*m DF was used. The GC oven program began at 40°C and was held for 2 min, followed by a gradual increase of 10°C/min till 250°C, then 20°C/min to 280°C, and a final hold for 10 min at 280°C. For GC-MS detection, an electron ionization system was operated in electron impact mode. Helium gas (99.9999%) was used as a carrier gas at a constant flow rate of 1 mL/min, and an injection volume of 1 *μ*L was employed. The injector temperature was maintained at 250°C and the ion-source temperature was 220°C. Mass spectra were taken at 70 eV, a scanning interval of 1 s, and fragments from 50 to 4500 Da. The solvent delay was from 0 to 3 min, and the total GC/MS run time was 34.5 min.

### 2.4. Compound Identification

The oil compositions were identified by matching their mass spectra data to the National Institute of Standard and Technology (NIST) and Wiley mass spectra databases, as well as mass spectra present in published data and confirmed by comparison of their relative retention indices with literature values. The retention indices, relative to C9–C40 n-alkanes, were obtained on GC-MS runs with the same conditions as that of the essential oils. The retention index was calculated using

(1) $$\begin{array}{c} \text{RI}=100n+100\left[\frac{\left(T_{\text{rx}}-T_{n}\right)}{\left(T_{\text{rx}}+1-T_{n}\right)}\right], \end{array}$$

where *T*_rx_ is the retention time of your target compound, *T*_*n*_ is the retention time of the n-alkane before your target compound, and *T*_rx+1_ is the retention time of the *n* + 1 alkane after the target peak. The quantitative composition of each peak was derived as percentages calculated by normalization of the peak area [19].

### 2.5. Antimicrobial Assay

#### 2.5.1. Media Preparation and Microbial Strains

Microbes used included 2 Gram-positive bacteria (*Staphylococcus aureus* ATCC 29213, and *Staphylococcus pneumoniae* ATCC 49619), 5 Gram-negative bacteria (*Bacillus subtilis*, *Salmonella typhi*, *Pseudomonas aeruginosa* ATCC 27853, *Klebsiella pneumoniae* ATCC 700603, and *Escherichia Coli* ATCC 25922,) and a fungus (*Candida albicans*). Stock cultures of bacterial strains were cultivated in a nutrient broth at 37°C for 24 h before the testing. A colony suspension in sterile saline was adjusted to 0.5 McFarland standard and further diluted in sterile double strength nutrient broth to obtain approximately ∼2 × 10^5^ CFU/mL.

#### 2.5.2. Minimum Inhibitory Concentrations (MIC) Determination

The minimum inhibitory concentrations of the essential oils were determined in the broth dilution assay. Two-fold serial dilution of the essential oil or standard antibiotic (ciprofloxacin) was prepared in sterile 96-well microtiter plates. In each plate, 100 *μ*L of two-fold serial dilution of essential oil was transferred into the wells. To each well was added 100 *μ*L of double strength nutrient broth containing an inoculum size of ∼2.0 × 10^5^ CFU/mL. After incubation for 24 hours at 37°C, 20 *μ*L of 1.25 mg/mL 3-(4, 5-dimethylthiazol-2-yl)-2,5-diphenyltetrazolium bromide solution (MTT) was added to each well and further incubated for 30 min at 37°C. The MIC was determined as the lowest concentration of essential oil or antibiotic that completely inhibited the growth of the organism in microdilution wells as detected by the absence of the purple coloration after MTT addition during a 24-hour incubation period at 37°C. All tests were performed in triplicate [20, 21].

### 2.6. Biofilm Inhibition

In the crystal violet staining assay, sterile microtiter plates were first filled with MIC and sub-MIC concentrations of essential oils and standard drug (gentamicin). Thereafter, a bacterial suspension adjusted to a turbidity of 0.5 McFarland standard was inoculated into each well. The final volume of each well was 200 *μ*L. A control experiment, in which solvent was used to replace essential oil, was also set up. The plates were incubated for 24 hours at 37°C and after that, the content of the wells was discarded followed by washing 3× with deionized water to remove loosely attached cells. The wells were then stained with 0.1% crystal violet followed by elution into a new plate for absorbance reading at 595 nm. Biofilm inhibition was estimated from the following equation:

(2) $$\begin{array}{c} \%\text{ inhibition}=\frac{A_{\text{c}}-A_{\text{t}}}{A_{\text{c}}}\times 100, \end{array}$$

where *A*_c_ is the absorbance of the control and *A*_t_ is the absorbance of the treatment groups [8].

### 2.7. Antioxidant Activity

The antioxidant activity of the fruit pulp and leaf essential oil of *Chrysophyllum albidum* was evaluated via the phosphomolybdenum, hydrogen peroxide scavenging, the 2,2-diphenyl-1-picrylhydrazyl (DPPH) radical scavenging, and inhibition of lipid peroxidation assays.

#### 2.7.1. Phosphomolybdenum Assay

In the phosphomolybdenum (PM) assay, essential oils of different concentrations were prepared in dimethyl sulfoxide (DMSO). Five milliliters of the PM reagent (0.6 M sulfuric acid, 28 mM sodium phosphate, and 4 mM ammonium molybdate) were added to 0.5 mL of each test sample in a test tube, shaken, and then incubated at 95°C for 90 min. After the reaction mixture had cooled to room temperature, the absorbance at 695 nm was read against a blank solution. Ascorbic acid was used as a standard drug in this experiment [22].

#### 2.7.2. Hydrogen Peroxide Scavenging Assay

The effective hydrogen peroxide scavenging activity of the essential oils was assayed by adding to a test tube ferrous ammonium sulfate (1 mM, 0.5 mL), followed by 0.13 mL of 5 mM H_2_O_2_ and 3 mL of essential oil or standard drug at varying concentrations. Each test tube was incubated in the dark for 5 min at room temperature. After this, 1,10-phenanthroline (1 mM, 3 mL) was added to each mixture and the test tube was shaken to ensure uniform mixing. The reaction mixture was then incubated at room temperature for 10 min. The absorbance of the reaction mixture was taken at 510 nm. Water was used in place of essential oils for the blank. Gallic acid was used as a standard drug. The amount of hydrogen peroxide scavenged was obtained from the following equation:

(3) $$\begin{array}{c} \%\text{ hydrogen peroxide scavenged}=\frac{A_{\text{test}}}{A_{\text{control}}}\times 100, \end{array}$$

where *A*_test_ is the absorbance of the test sample and *A*_control_ is the absorbance of the blank [23].

#### 2.7.3. DPPH Radical Scavenging Assay

The scavenging activity of 2,2-diphenyl-1-picrylhydrazyl (DPPH) free radicals of fruit pulp and leaf essential oil of *Chrysophyllum albidum* was performed according to the method used by Gyesi et al. [11]. Methanol was used as the negative control. L-Ascorbic acid was used as a standard drug. The percent inhibition of DPPH free radicals was calculated from the absorbance of the control (*A*_c_) and of the test (*A*_t_) from the following equation:

(4) $$\begin{array}{c} \text{inhibition}\left(\text{\%}\right)=\left[\frac{A_{\text{c}}-A_{\text{t}}}{A_{\text{c}}}\right]\times 100. \end{array}$$

#### 2.7.4. Inhibition of Lipid Peroxidation (TBARS Assay)

This assay was performed according to methods described by Damien Dorman et al. with slight modification [24]. Egg yolk rich in lipids was used as a source of an oxidable substrate. Briefly, 0.5 mL of 10% (w/v in 1.5% KCl) homogenate and 0.1 mL of sample solubilized in methanol were added to a test tube and made up to 1.0 mL with distilled water. An aliquot of 50 *μ*L of FeSO_4_ (0.07 M) was added to each sample to induce lipid peroxidation. To the mixture, 1.5 mL of 20% trichloroacetic acid (TCA) and 0.8% (w/v) thiobarbituric acid (TBA) in 1.1% (w/v) sodium dodecyl sulfate (SDS) stock reagent was added and then 1.5 mL of 20% acetic acid (pH adjusted to 3.5 with NaOH) was added. The resulting mixture was vortexed, followed by heating at 95°C for 60 min. After cooling, 5.0 mL of butanol was added to each tube and centrifuged at 3000 rpm for 10 min. The absorbance of the organic upper layer was measured at a wavelength of 532 nm. Butylated hydroxytoluene (BHT) and ascorbic acid were used as positive controls. The percentage inhibition of lipid peroxidation was calculated from the absorbance obtained using the following equation:

(5) $$\begin{array}{c} \%\text{ inhibition}\frac{A_{\text{c}}-A_{\text{s}}}{A_{\text{c}}}\times 100, \end{array}$$

where *A*_c_ is the absorbance of blank and *A*_s_ is the absorbance of the sample mixture.

The IC_50_ values (extract concentration needed to achieve 50% inhibition of lipid peroxidation) were obtained from a graph of % inhibition against concentration.

### 2.8. Data Analysis

All experiments were conducted in triplicates and data presented as mean ± standard deviation. Statistical analyses were performed in GraphPad Prism 6.0 for Windows (GraphPad Software, San Diego, CA, USA). *p* < 0.05 was considered to be statistically significant.

## 3. Results

**Figure 1:**
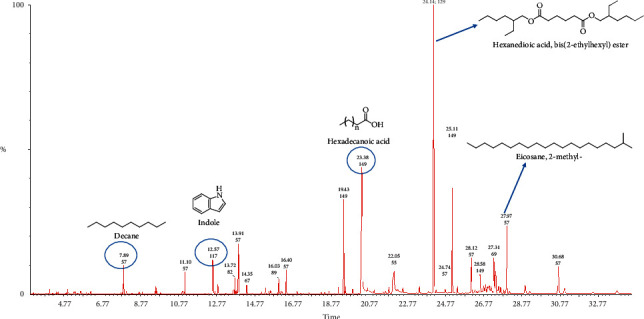
Total ion chromatogram (TIC) obtained from the GC-MS run of the essential oil from the leaves of *Chrysophyllum albidum.* Compounds were identified by comparison of MS spectra data with NIST and Wiley libraries as well as published literature. Chemical structures of some of the peaks are shown and were drawn with ChemDraw.

Steam distillation was used in isolating essential oils from fruits and leaves of *Chrysophyllum albidum* in yields of 0.53% and 0.17%, respectively. Both essential oils were pale yellow in color. Figures 1 (leaf) and 2 (fruit) represent the total ion chromatograms obtained from the GC-MS analysis. Compounds were identified by comparing the mass spectra obtained in the GC-MS run with available NIST and Wiley spectra libraries. Confirmation of identified constituents was done by comparing the computed linear retention index of each compound to corresponding indices in literature and available web-based sources [19, 25]. A total of 35 compounds were identified in the leaf essential oil and this represented 97.84% of the total oil constituents. For the fruit essential oil, 34 compounds which represent 97.86% of the total oil constituents were identified. Tables 1 and 2 show the chemical composition of the fruit and leaf essential oils. The leaf essential oil was made up of compounds classified as alkanes (∼28.49%), carboxylic acids (∼28.38%) esters (∼27.87%), and terpenes/terpenoids (∼5.55%). The fruit essential oil also had alkanes (∼26.90%), esters (∼26.29%), and terpenes/terpenoids (∼24.25%) as the most abundant constituents (Table 3).

The essential oils from the fruit and leaf of *Chrysophyllum albidum* were screened against 8 bacteria and 1 fungus. The results of the antimicrobial susceptibility test are represented in Table 4. The MICs of the leaf essential oil against all tested microorganisms ranged between 6.875 and 13.750 mg/mL, whereas those of the fruit essential oils were between 0.195 and 6.250 mg/mL. In general, the fruit essential oil exhibited better antimicrobial activity when compared to the leaf essential oil. *Bacillus subtilis*, *Salmonella typhi*, and *Pseudomonas aeruginosa* were the most susceptible organisms to the fruit essential oil with MICs less than 2 mg/mL, whereas *Candida albicans, Staphylococcus aureus,* and *Pseudomonas aeruginosa* were the most susceptible organisms to the leaf essential oil. Both essential oils displayed medium antimicrobial activity towards *Candida albicans,* which was the only fungus used in this study.

In the biofilm inhibitory assays, the fruit and leaf essential oils were screened against *Pseudomonas aeruginosa* biofilms. Essential oils were evaluated at MIC and sub-MICs (Table 5). Interestingly for both extracts, there was over 50% biofilm inhibition at all concentrations tested. For both extracts, a maximum inhibitory effect of over 70% was recorded. The maximum biofilm inhibitory effect of the positive control, gentamicin, was 88.42% at its MIC. In general, there was a dose-dependent relationship in biofilm inhibition for all treatments. The concentration of leaf essential oil required to elicit half-maximal inhibition of biofilm formation (BIC_50_) was significantly different (*p* < 0.05) from that of the fruit essential oil. The fruit essential was much better at inhibiting biofilm formation (BIC_50_ of 4.78 ± 0.21 mg/mL) than the leaf essential oil (BIC_50_ of 6.97 ± 0.56 mg/mL). Gentamicin was, however, superior to both leaf and fruit essential oils with a BIC_50_ of 1.62 ± 0.28 *μ*g/mL.

In the phosphomolybdenum assay, the total antioxidant capacity for the leaf and fruit essential oils was determined to be 104.76 ± 2.4 and 101.59 ± 0.83 *μ*g/g AAE, respectively. There was no significant difference (*p* < 0.05) in the TAC of the two essential oils. The IC_50_ values obtained in the hydrogen peroxide scavenging test were 1048 ± 0.27 and 1454 ± 0.26 *μ*g/mL for the leaf and fruit essential oil, respectively. Gallic acid was used as the standard in the hydrogen peroxide scavenging test. Comparatively ascorbic acid performed better than both the leaf and fruit essential oils with an IC_50_ value of 87.51 ± 0.37 *μ*g/mL. Based on the recorded IC_50_ values, the leaf essential oil was about 50% better at scavenging hydrogen peroxide when compared to the fruit essential oil. Both oils though can be described as possessing only moderate activity in this assay. The IC_50_ values for the leaf and fruit essential oils in the DPPH assay were 301.8 ± 0.7 and 669.2 ± 2.12 *μ*g/mL, respectively. Ascorbic acid, with an IC_50_ of 11.94 ± 1.9 *μ*g/mL, was used as a standard drug. A dose-dependent scavenging of the DPPH radical was observed for all test and standard drugs. The leaf and fruit pulp essential oil showed good activities in the inhibition of lipid peroxidation test. The concentrations at which 50% of lipid peroxidation was inhibited were determined to be 460.1 ± 2.7 and 457.4 ± 0.3 *μ*g/mL for the leaf and fruit essential oils. The IC_50_ value for butylated hydroxytoluene (BHT), a standard drug, was 8.0 ± 0.4 *μ*g/mL.  Table 6 summarizes the results for the antioxidant activities of the leaf and fruit pulp essential oil of *Chrysophyllum albidum*.

## 4. Discussion

Many plant metabolites have served as lead compounds in the discovery of drugs [26]. Essential oils, so-called, because they represent the essence of plants, are mostly obtained by steam distillation, hydrodistillation, and cold pressing of various plant parts. Essential oils have been shown to possess broad-spectrum biological effects and hence they have been investigated for these purposes. Essential oils are mostly present in trace amounts in plant epidermic cells, secretory cells, canals, and cavities. The diverse compounds present in essential oils contribute to the aroma of the oil [27]. The major compound present in an essential oil may range from 20 to 85%. These major constituents may contribute greatly to the aroma and biological activity of the essential oil. Compounds present in trace amounts may also act in synergy to elicit specific biological effects [28]. The low amounts of essential oils secreted by plants result in the low yield of these oils after extraction. Mostly, the yield of essential oils reported is less than 1% [28] and the yields obtained from the leaf and fruits (0.17% and 0.53%, resp.) of *Chrysophyllum albidum* follow this trend.

To identify the chemical constituents of essential oils, GC-MS is normally employed. Characterization of essential oil constituents could serve as a fingerprint for that plant and also help in identifying the chemical constituents that may be responsible for any observed biological activities. The essential oil of the leaf was dominated by hydrocarbons, carboxylic acids, and terpenes. n-Hexadecanoic acid was the major compound present in the leaf essential oil and constituted 20.25% of the total oil composition. The nature of this major component, which is a fatty acid, suggests it may act as an antioxidant [29]. A previous study by Ishola identified the sesquiterpene, *α*-farnesene (38.11%), as the major compound present in the leaf essential oil [18]. In this study, *α*-farnesene represented only 0.56% of the total leaf essential oil composition. The fruit pulp essential oil was dominated by terpenoids, hydrocarbons, and esters. Hexanedioic acid, bis(2-ethylhexyl) (16.26%), was the major compound present in the fruit pulp essential oil. Lasekan and coworkers utilized a headspace solid-phase microextraction and GC-MS to determine the volatile constituents of the pulp of the fruit in attempt to identify compounds which contribute to the aroma of fruit juice made from the African star apple. Esters (such as ethyl dodecanoate, methylhexanoate, ethyl hexanoate, and hexyl butanoate) and terpenoids (such as *α*- and *β*-pinene, geraniol, and *α*-muurolene) were the major contributors to the aroma of the fruit juice [17]. Esters in essential oils are usually the products of the esterification of alcohols and acyl-CoAs derived from both amino acid and fatty acid metabolisms in the plant [30]. In Ishola's study, essential oils from the fruit bark of the *Chrysophyllum albidum* plant was also studied [18]. Not surprisingly, some compounds present in the essential oil from the pulp were also identified in the fruit bark essential oil. One such common compound is à-amyrin. In general, the differences in the chemical constituents of the leaf and fruit essential oil analyzed in this study and those of other previous works may be attributed to geographical variations, stage of fruit or leaf maturity, nutritional condition of the plant, as well as chemotypic variations in the plants used [31, 32].

Essential oils have gained much interest in the food industry due to their potential applications as natural food preservatives against food spoilage microbes [33]. The fruit and leaf essential oils exhibited broad-spectrum antimicrobial activity against all tested pathogens. Overall, the fruit essential oil exhibited better antimicrobial activity as compared to the leaf essential oil (Table 4). The fruit essential oil showed better activity against *Bacillus subtilis*, *Salmonella typhi*, and *Pseudomonas aeruginosa*. Ishola and team had earlier shown that some strains of *Escherichia coli*, *Bacillus subtilis*, *Salmonella typhi,* and *Pseudomonas aeruginosa* were resistant to the leaf essential oil but *Staphylococcus aureus* was susceptible. A different trend was observed in this study, where the leaf essential oil demonstrated broad-spectrum antimicrobial activity against *Escherichia Coli*, *Bacillus subtilis*, *Salmonella typhi,* and *Pseudomonas aeruginosa*. The differences in the antimicrobial activity of the leaf essential oil in this study and that of the Nigerian study may be as a result of differences in chemical compositions of the leaf essential oils in the 2 studies. The leaf essential oil in this study contains essential components such as linalyl anthranilate, which might be responsible for the observed antimicrobial activity. This compound was absent in the study by Ishola and team. Linalyl anthranilate has been proposed to react with bacteria cell membrane components to produce reactive oxygen species (ROS). These ROS could initiate lipid oxidation via a membrane lipid attack and could thus disrupt bacterial cell membrane and hence leak intracellular contents. The ROS could also leak into intracellular regions and degrade nucleic acids, lipids, and/or proteins [34]. The presence of sesquiterpenes may also contribute to the antimicrobial activity of essential oils. Sesquiterpenes, due to their inherent lipophilicity properties, could enhance an intracellular accumulation of DNA, and thus impermeabilizing the cytoplasmic membrane of *Lactobacillus fermentum*. This suggests that the increased permeability will potentiate the rupture of the cytoplasmic membrane [35]. In both fruit pulp and leaf essential oils, the sesquiterpenes, à-amyrin, and (Z, E)-*α*-farnesene were present, respectively (Tables 1 and 2). These molecules may as well contribute to the antimicrobial activity of the essential oils.

*Pseudomonas aeruginosa* is a biofilm-forming organism [8]; hence the activity of the oils against this organism prompted us to investigate the biofilm inhibition potential of the fruit and leaf essential oil of *Chrysophyllum albidum*. Both fruit and leaf essential oil exhibited very good inhibitory properties, with percentage inhibition above 50% at all concentrations tested (Table 5). The fruit essential oil demonstrated good antibiofilm activity with a BIC_50_ value of 698.80 *μ*g/mL as opposed to the leaf essential oil with a BIC_50_ value of 477.9 *μ*g/mL. Biofilm formation usually occur in 4 phases: initial surface attachment of bacteria cells, microcolony formation through assembly of the bacteria cells, biofilm maturation, and, finally, biofilm dispersal. Abolishing the initial attachment of planktonic cells to surfaces has been suggested as a promising route for antibiofilm activity [36]. Essential oil constituents such as eugenol and the amyrins have been shown to disrupt initial attachment of bacteria cells to various surfaces and thus interfere with biofilm formation [37, 38]. The presence of these compounds in both essential oils investigated in this work may suggest a similar mode of antibiofilm action. Essential oils could interfere with quorum sensing factors produced by Gram-negative and Gram-positive bacteria. Quorum sensing mediates virulence factors production, swarming motility, and biofilm formation in many bacteria [8]. Thus, interrupting the action of quorum sensing factors could also lead to antibiofilm action. Terpenoids such as eugenol and geraniol have been shown to also possess anti-quorum sensing properties and thus inhibit biofilm formation via this route [39].

Oxidation is a relevant biochemical process, but complete oxidation is very detrimental to the health status of living organism because it can cause damage to cell components, including DNA, lipids, and proteins [40]. Scavenging and subsequent neutralization of species which can promote excessive oxidation is thus important. The essential oils demonstrated free radical scavenging activity in the DPPH and H_2_O_2_ assays. For the DPPH radical scavenging assay, the leaf and fruit pulp essential oils were able to scavenge 50% of free radical at concentrations of 301.8 ± 0.68 and 669.2 ± 2.12 *μ*g/mL. Comparatively, the leaf essential oil acted as better hydrogen donating or radical scavenging activity in the DPPH assay. In the hydrogen peroxide assay, the leaf essential oil performed superiorly with an IC_50_ value of 1048 ± 0.27 *μ*g/mL as compared to the fruit pulp essential oil with an IC_50_ value of 1454 ± 0.26 *μ*g/mL. Sterols and esters contribute to the antioxidant activities of fruit essential oil. Gyesi and team investigated the chemical composition and antioxidant activity of the fruit pulp essential oil of *Annona muricata.* The fruit pulp essential oil of *Annona muricata* reveals esters and sterols as major compounds present in the oil. Fatty acids, sterols, and esters are known to contribute to antioxidant activity [11]; hence the high content of esters and fatty acids in the fruit pulp and leaf essential oils may have contributed to the antioxidant activity in the present study. Also, the presence of compounds such as eugenol, muurol-5-en-4-alpha-ol, davanone-2-ol, and phytol in the fruit pulp essential oil may contribute to the antioxidant activity. The total antioxidant capacities for both fruit pulp and leaf essential oils were almost similar. To scavenge ROS, the antioxidant agent could interfere with the initiation or chain propagation steps involved in free radical-mediated oxidation. The antioxidant agent could also induce processes that lead to the production of antioxidant enzymes [41]. Lipid peroxidation leads to spoilage of lipid-rich foods. Inhibition of lipid peroxidation in foods is thus very desirable in potential food additives. The low IC_50_ of the leaf and fruit pulp essential oils in the inhibition of lipid peroxidation assays is an indication that these oils could be harnessed as preservatives. In recent times, the use of synthetic antioxidants such as BHT and BHA has been met with a negative perception of consumers, hence the need for safe and naturally derived antioxidants that can be applied in the food industry [33]. The fruit and leaf essential oils of *Chrysophyllum albidum* have shown good antioxidant activities in a variety of assays and could potentially have a variety of applications in the food industry.

## 5. Conclusion

The study evaluated the chemical composition and some biological properties of the fruit and leaf essential oils of *Chrysophyllum albidum.* Thirty-five compounds were identified in the leaf essential oil, with n-hexadecanoic acid as the most abundant compound. In the fruit essential oil, 34 compounds were identified. The oils demonstrated broad-spectrum antimicrobial activity against some pathogenic microorganisms and impressive biofilm inhibition properties against *Pseudomonas aeruginosa.* All oils also showed very good antioxidant potential and inhibited lipid peroxidation. Based on the antioxidant and antimicrobial activities of the fruit and leaf essential oil of *Chrysophyllum albidum,* the oils could have potential applications as food preservatives.

## Figures and Tables

**Figure 2:**
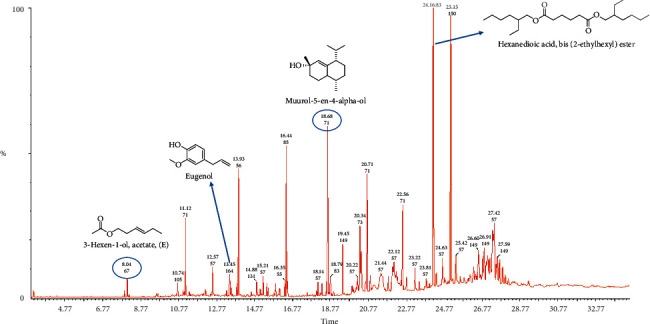
Total ion chromatogram (TIC) obtained from the GC-MS run of the essential oil from the fruits of *Chrysophyllum albidum*. Compounds were identified by comparison of MS spectra data with NIST and Wiley libraries as well as published literature. Chemical structures of some of the peaks are shown and were drawn with ChemDraw.

**Table 1:** Chemical composition of the leaf essential oil of *Chrysophyllum albidum* as determined by GC-MS.

S/N	Compound name	% C	RI^a^	RI^b^
1	1,3-Dimethylcyclohexane cis-trans	0.375	740	^*∗*^ni
2	Ethylcyclohexane	0.473	810	^*∗*^ni
3	4,6-Octadiyn-3-one, 2-methyl-	0.434	846	^*∗*^ni
4	Decane	1.720	972	1000
5	Linalyl anthranilate	0.894	1071	^*∗*^ni
6	Undecane	2.097	1166	1100
7	Indole	3.419	1363	1301
8	Ethanone, 1-(2-hydroxy-5-methylphenyl)-	0.558	1381	1316
9	cis-Hexanoic acid	0.415	1341	^*∗*^ni
10	2,7-Octadiene, 4-methyl-	1.051	1345	^*∗*^ni
11	Tetradecane	3.460	1358	^*∗*^ni
12	1,5-Cyclooctadiene, 1,3-dimethyl-	0.745	1390	^*∗*^ni
13	(Z,E)-*α*-farnesene	0.556	1465	1488
14	3,7,11-Trimethyl-3-hydroxy-6,10-dodecadien-1-yl acetate	1.377	1520	^*∗*^ni
15	3-Hexen-1-ol, benzoate, (Z)-	0.388	1529	1569
16	Hexadecane	1.555	1550	1600
17	Sulfurous acid, 2-ethylhexyl hexyl ester	0.380	1740	^*∗*^ni
18	Hexahydrofarnesyl acetone	0.416	1786	1801
19	Farnesyl acetate <(Z,E)->	6.210	1813	1818
20	4-Benzyloxybenzoic acid	0.644	1823	^*∗*^ni
21	n-Hexadecanoic acid	20.245	1903	1942
22	1-(Hydroxymethyl)-1-(2′-hydroxyethyl) cyclopropane	0.388	1927	^*∗*^ni
23	2,7-Dimethyloctane	1.634	1932	^*∗*^ni
24	Phytol	0.574	2046	2096
25	Alkynyl stearic acid	6.905	2073	^*∗*^ni
26	Undecanoic acid	1.230	2092	^*∗*^ni
27	Hexadecane	0.396	2124	^*∗*^ni
28	2-Bromotetradecane	0.473	2218	^*∗*^ni
39	Hexanedioic acid, bis(2-ethylhexyl) ester	19.100	2307	^*∗*^ni
30	Tridecanol, 2-ethyl-2-methyl-	1.081	2391	^*∗*^ni
31	2-Bromononane	0.461	2494	^*∗*^ni
32	1-Iodo-2-methylundecane	4.077	2632	^*∗*^ni
33	Squalene	4.097	2193	ni
34	Eicosane, 2-methyl-	7.876	2275	ni
35	2-methyloctacosane	4.289	2386	2388

S/N: compound number in order of elution; %C-% composition of compound in essential oil; RI^a^: retention index calculated from retention times relative to C8-C40 n-alkanes on a DB-5 column; RI^b^: retention index from literature and web-based sources [19, 25]; ^*∗*^ni-not identified (compounds do not have their retention index reported for a DB-5 column in literature; compound identified from the NIST or Wiley database by comparing mass spectra with spectral similarity score of 70 and above.

**Table 2:** Fruit essential oil composition of *Chrysophyllum albidum* as determined by GC-MS.

S/N	Compound name	% C	RI^a^	RI^b^
1	3-Hexen-1-ol, acetate, (E)	0.886	980	1018
2	Benzoic acid, ethyl ester	1.043	1142	1165
3	Cyclododecane	0.524	1159	^*∗*^ni
4	Dodecane	4.151	1167	^*∗*^ni
5	Tridecane, 7-methy	0.511	1337	^*∗*^ni
6	Eugenol	1.824	1325	1352
7	7-Hexadecene, (Z)	0.873	1300	^*∗*^ni
8	Tetradecane	8.314	1359	^*∗*^ni
9	2-Propenoic acid, 3-phenyl-, ethyl ester	1.029	1429	1462
10	1-Cyclohexene, 1,3,3-trimethyl-2-(1-methylbut-1-en-3-on-1-yl	0.822	1471	^*∗*^ni
11	1,1,4,5,6-pentamethyl-2,3-dihydro-1H-indene	0.444	1477	^*∗*^ni
12	Pentadecane, 3-methyl	0.561	1523	^*∗*^ni
13	1-Hexadecanol	0.909	1545	1873
14	Muurol-5-en-4-alpha-ol	11.478	1553	1554
15	Davanone-2-ol	0.640	1717	1717
16	Sinensal	10.140	1745	1751
17	Eicosane	8.263	1851	^*∗*^ni
18	n-Hexadecanoic acid	4.256	1936	1961
19	Manool (epi-13)	1.089	1951	1958
20	Methyl 3,5-dicyclohexyl-4-hydroxybenzoate	1.422	1975	^*∗*^ni
21	1,4-Epoxynaphthalene-1(2H)-methanol,4,5,7-tris(1,1 dimethylethyl)-3,4-dihydro	6.083	2010	^*∗*^ni
22	Phytol	0.916	2048	2096
23	11,14-Eicosadienoic acid, methyl ester	3.362	2073	^*∗*^ni
24	Stearic acid	1.687	2092	2169
25	Heneicosane, 3-methyl	1.813	2100	^*∗*^ni
26	9-Tricosene, (Z	1.367	2191	^*∗*^ni
27	Hexanedioic acid, bis(2-ethylhexyl) ester	16.264	2309	^*∗*^ni
28	Octadecane, 3-ethyl-5-(2-ethylbutyl	0.526	2404	^*∗*^ni
39	à-Amyrin	0.812	2579	^*∗*^ni
30	Oleyl palmitoleate	1.085	3360	^*∗*^ni
31	1,2-Propanediol, 3-(octadecyloxy)-, diacetate	0.504	3446	^*∗*^ni
32	Oleic acid, 3-(octadecyloxy)propyl ester	0.902	3507	^*∗*^ni
33	tri(2-Ethylhexyl) trimellitate	1.904	3539	^*∗*^ni
34	7,8-Epoxylanostan-11-ol, 3-acetoxy	1.115	3577	^*∗*^ni

S/N: compound number in order of elution; %C-% composition of compound in essential oil; RI^a^: retention index calculated from retention times relative to C8-C40 n-alkanes on a DΒ-5 column; RI^b^: retention index from literature and web-based sources [19, 25]; ni: not identified (compounds do not have their retention index reported for a DB-5 column in literature); compound identified from the NIST or Wiley database by comparing mass spectra with spectral similarity score of 70 and above.

**Table 3:** Classification of compounds identified in both leaf and fruit essential oils of *Chrysophyllum albidum*.

Compound class	Leaf essential oil (% composition)	Fruit essential oil (% composition)
Alcohols	1.08	2.97
Alkanes	28.49	26.90
Carboxylic acids	28.38	7.03
Esters	27.87	26.29
Ketones	0.97	1.03
Sterols	0.57	0.92
Terpenes/terpenoids	5.54	24.25
^#^Others	4.92	8.46
Total	97.84	97.86

^#^Others include compound classes such as aromatic hydrocarbons, coumarins, amines, and phenols.

**Table 4:** Antimicrobial activity: minimum inhibitory concentrations of fruit and leaf essential oils of *Chrysophyllum albidum* against various pathogenic microorganisms.

Microorganism (Gram status)	Minimum inhibitory concentrations (mg/mL)		
	Leaf essential oil	Fruit essential oil	^*∗*^Ciprofloxacin
*S. aureus* (+)	6.875	6.250	1.563
*S. pneumoniae* (+)	13.750	6.250	3.125
*B. subtilis* (−)	13.750	0.195	3.125
*S. typhi* (−)	13.750	0.783	1.563
*P. aeruginosa* (−)	6.875	1.563	1.563
*E. coli* (−)	13.750	6.250	3.125
*K. pneumoniae* (−)	13.750	6.250	1.563
^*#*^ * C. albicans*	6.875	6.250	1.563

^*∗*^MICs of ciprofloxacin are in *μ*g/ml; +, Gram-positive bacteria; −, Gram-negative bacteria; ^#^*C. albicans* is a fungus.

**Table 5:** Inhibition of *P. aeruginosa* biofilm formation by of leaf and fruit essential oils of *Chrysophyllum albidum*.

^#^Concentrations	% biofilm inhibition		
	Leaf essential oil	Fruit essential oil	^*∗*^Gentamicin
MIC	72.96 ± 0.7	72.4 ± 1.2	88.42 ± 0.86
MIC/2	69.04 ± 0.9	55.39 ± 2.4	65.00 ± 1.21
MIC/4	68.76 ± 0.3	55.82 ± 2.3	57.07 ± 1.67
MIC/8	67.07 ± 0.9	55.18 ± 0.2	56.43 ± 1.34
MIC/16	65.13 ± 2.7	51.57 ± 0.4	48.91 ± 0.89
MIC/32	64.47 ± 0.4	50.60 ± 0.9	45.50 ± 1.10
^*∗∗*^BIC_50_ (mg/mL)	^*∗∗*^6.97 ± 0.56^a^	^*∗∗*^4.78 ± 0.21^b^	^*∗∗*^0.00162 ± 0.28^c^

Data presented as mean ± standard deviation, *n* = 3; ^#^concentrations of essential oils or gentamicin used were based on their minimum inhibitory concentration (MIC) against *P. aeruginosa*; ^*∗*^gentamicin was used for comparison purposes. ^*∗∗*^BIC_50_ values computed from dose-response curve. Different superscript letters (^a, b, c^) indicate significant difference in BIC_50_ values (*p* < 0.05).

**Table 6:** Antioxidant activities of leaf and fruit essential oils of *Chrysophyllum albidum* as determined from different assays.

Sample	Total antioxidant capacity (*μ*g/g AAE)	DPPH radical scavenging activity IC_50_ (*μ*g/mL)	H_2_O_2_ scavenging activity IC_50_ (*μ*g/mL)	^*∗∗*^TBARS IC_50_ (*μ*g/mL)
Leaf	104.8 ± 2.4	301.8 ± 0.7	1048.0 ± 0.3	460.1 ± 2.7
Fruit	101.6 ± 0.8	669.2 ± 2.1	1454.0 ± 0.3	457.4 ± 0.3
Ascorbic acid	nd	11.9 ± 1.9	nd	6.7 ± 0.3
Butylated Hydroxytoluene (BHT)	nd	nd	nd	8.0 ± 0.4
Gallic acid	nd	nd	87.5 ± 0.4	nd

Data represented as mean ± standard deviation, *n* = 3; ^*∗*^TAC: total antioxidant capacity; ^*∗∗*^TBARS: thiobarbituric acid reactive substance assay; nd: not determined (compound not used in that experiment).

## Data Availability

All the data generated or analyzed during this study are included in this published article.
